# Life Cycle Assessment of Low-Cost Membrane Bioreactor and Activated Sludge Systems for Decentralized Wastewater Treatment in Arid Regions

**DOI:** 10.3390/membranes16020074

**Published:** 2026-02-22

**Authors:** Husnain Haider, Md. Shafiquzzaman, Saleem S. AlSaleem, Abdul Razzaq Ghumman

**Affiliations:** Department of Civil Engineering, College of Engineering, Qassim University, Buraydah 51452, Saudi Arabia; h.chaudhry@qu.edu.sa (H.H.); sa.alsaleem@qu.edu.sa (S.S.A.); a.muhammed@qu.edu.sa (A.R.G.)

**Keywords:** sustainability, environmental impacts, life cycle assessment, decentralized wastewater treatment, smaller communities, arid regions

## Abstract

Small communities in the Kingdom of Saudi Arabia (KSA) without a sewerage system commonly rely on septic tanks and long-distance transport of wastewater to the nearest centralized treatment facilities, resulting in high operational costs, social nuisance, and limited opportunities for treated effluent reuse. For a small community of 1300 persons in Al Qaraa (Qassim, KSA), this study performs life cycle analysis (LCA) to evaluate the environmental sustainability of a low-cost membrane bioreactor (LC-MBR)-type for decentralized on-site treatment as an alternative to wastewater transportation to a conventional extended aeration activated sludge process (EA-ASP)-type centralized system operating in the nearest larger city of Al-Bukayriyah. SimaPro^®^ 8.3.0.0 with the ecoinvent 3.0 database and ReCiPe 16 midpoint methodology shows that the decentralized LC-MBR scenario outperformed the centralized option with a 49 km-long wastewater transportation route in 13 out of 15 selected midpoint categories when considering relative and normalized impacts. In the EA-ASP, primary treatment dominated environmental impacts across most categories, driven by high energy demand for wastewater pumping, whereas freshwater and marine eutrophication were primarily influenced by treatment efficiency. With smaller normalized values, secondary treatment had a greater relative impact on urban and agricultural land occupation categories, attributed to the use of clay and rice bran in low-cost membrane fabrication in an LC-MBR. Tertiary treatment in the LC-MBR scenario, incorporating coagulation and granular activated carbon, significantly reduced freshwater eutrophication. Although normalized endpoint impacts indicated comparable ecosystem impacts for both systems, the LC-MBR resulted in 8% lower impacts on human health and 60% lower on resource depletion. Overall, the findings support decentralized wastewater treatment as a sustainable solution for small communities in arid regions and provide valuable insights for policy and decision-making.

## 1. Introduction

In the Kingdom of Saudi Arabia (KSA), wastewater reclamation has been recognized as a critical focus area due to the rapid increase in urban water demand and the environmental and resource-conservation benefits of treating and reusing municipal wastewater. The idea of wastewater reuse is increasingly relevant in urban settings that rely primarily on desalinated water in arid regions. Most of the treated effluent (i.e., ~70% of total production) from centralized wastewater treatment plants in KSA’s cities is reused for urban landscaping and restricted or unrestricted irrigation; however, it is discharged into natural drains and rivers during the rainy season [1,2].

Wastewater reuse at the point of generation, with minimal financial and environmental impacts, is an indicator of sustainable communities [3]. In the absence of a sanitary sewerage network, domestic sewage from septic tanks must be transported to the nearest centralized wastewater treatment (CWWT) plant, creating multiple challenges for smaller communities and rural areas in KSA. First, wastewater transportation may limit the community’s opportunities to benefit from the sustainable reuse of treated wastewater, particularly in arid regions. Secondly, this on-site wastewater handling option poses various socio-economic and environmental challenges for residents [4]; for instance: (i) inconvenience caused by calling and waiting for collection trucks after every two to three weeks; (ii) nuisance caused by noise and emissions generated from sewage pumping and vehicle movement; and (iii) economic burden for bearing the cost of septic tank’s desludging and sewage transportation [5].

Decentralized wastewater treatment (DWWT) plants can help mitigate the challenges mentioned above and provide a sustainable wastewater management solution for smaller communities in KSA. A low-cost ceramic membrane bioreactor (LC-MBR) made of locally available materials can economically replace conventional biological membranes for wastewater treatment. Authors successfully employed the LC-MBR to treat sand-filter backwash water [6], ablution water [7], and stormwater [8] in KSA. In 2023, Alresheedi et al. [9] developed an LC-MBR in the Environmental Engineering Laboratory of the College of Engineering at Qassim University as a decentralized wastewater treatment option for smaller communities in KSA. The LC-MBR effluent met the irrigation reuse standards recommended by KSA and the World Health Organization [10,11]. A subsequent study by the authors of [5] employed a resident-perception-based approach to assess the willingness to adopt the LC-MBR system for decentralized wastewater treatment, revealing strong potential for this sustainable, community-managed technology, particularly when supported by initial government intervention.

Most centralized facilities in KSA use the extended aeration activated sludge process (EA-ASP), which has much higher energy requirements, particularly for aeration, compared to a membrane-based LC-MBR [12]. Wastewater transportation further increases energy footprint of the current scenario for smaller communities in KSA. The manufacturing of ceramic membranes can contribute to the overall energy demand of the DWWT wastewater treatment and reuse scenario at the point of generation [13]. Although DWWT appears to be a more promising option from a socio-economic perspective, a detailed life cycle assessment (LCA) is essential to help decision-makers evaluate the environmental impacts of both the baseline CWWT system and the proposed DWWT reuse scenario, and to select the most sustainable approach for smaller communities in KSA.

Past studies have frequently employed LCA-based approaches to evaluate the environmental performance of wastewater treatment facilities in both developing and developed countries [14]. Kobayashi et al. [15] performed LCA to compare constructed wetlands and membrane bioreactors (MBR) as decentralized greywater treatment systems with conventional centralized WWT for various reuse combinations of irrigation, toilet flushing, and laundry. They used a 50-year lifespan for the treatment system and ecoinvent 3.0 for background data inventory. OpenLCA with TRACI 2.1 was adopted for life cycle impact assessment (LCIA). They adopted Cascadia Green Building Council [16] data on materials and chemicals used in the construction and operation of the MBR and ignored landfill emissions due to a lack of data. Their study highlighted the conventional MBR as the most environmentally preferable option for greywater treatment in a community of 3500 persons equivalent (PEI).

Using SimaPro^®^ 8.3.0.0, the ecoinvent 3.0 database, and the ReCiPe 16 midpoint methodology, Mamathoni and Harding [17] reported that EA-ASP had overall higher environmental impacts than a simpler sequential batch reactor. The batch reactors evaluated in their study were equipped with surface aerators to oxidize dissolved and suspended waste biologically. Similar to MBRs, the sludge settles and is removed from the same reactor. The findings support the use of simplified wastewater treatment technologies for developing countries. Risch et al. [18] compared a conventional ASP-type centralized WWT system with a vertical-flow constructed wetland (CW) followed by sand filters as the decentralized treatment system. They found the ASP showed fewer impacts on the ecosystem endpoint category due to more controlled effluent discharges. In contrast, the decentralized option was found to be more sustainable than the conventional ASP in the resource endpoint category. Interestingly, wetlands completely surpassed the ASP in all three endpoint categories when applied to a smaller community.

Lourenço and Nunes [19] conducted an LCA of vermifilteration, CW, and ASP for decentralized wastewater treatment in small communities in southern Europe. Their study found vermifiltration to be more environmentally sustainable, with lower impacts in most categories during the operation phase, compared to CW and the ASP, particularly for smaller communities. Gallagher and Gill [20] performed LCA to compare the environmental performance of septic tank systems, packaged treatment units, and a willow evapotranspiration system for decentralized wastewater treatment in Ireland. Their studies found that evapotranspiration stems have the lowest environmental impacts. Juem et al. [21] performed LCA of a wastewater treatment plant in the United Arab Emirates (UAE) using OpenLCA. They identified global warming, human toxicity, ecotoxicity, and eutrophication as the major categories, and wastewater transport and material production as the main contributors to environmental impacts.

The review of past studies highlights three important findings. First, the use of simplified technologies for decentralized wastewater treatment poses a lower environmental burden in smaller communities. Second, limited LCA studies on conventional polymeric membrane bioreactors are available. Third, no detailed LCA study is available for smaller communities in arid regions that compares an EA-ASP-type centralized wastewater treatment option (with wastewater transportation from the community to the plant) with decentralized low-cost membrane bioreactor treatment systems manufactured from local materials.

The present study aims to conduct an LCA of an LC-MBR-type decentralized wastewater treatment scenario for smaller communities in KSA and of the existing wastewater transportation scenario to the nearest CWWT facility. The findings will highlight key impacts associated with the treatment technologies and identify priority areas for improving environmental performance. The study’s insights will help municipalities and concerned organizations, such as the National Water Company (NWC), develop more reliable and environmentally friendly WWTP designs. The findings will also provide stakeholders with more robust environmental information to support strategic planning and achieve long-term sustainability objectives of the water sector in KSA and other arid regions with similar geographical and social settings.

## 2. Materials and Methods

### 2.1. Study Region

**Figure 1:**
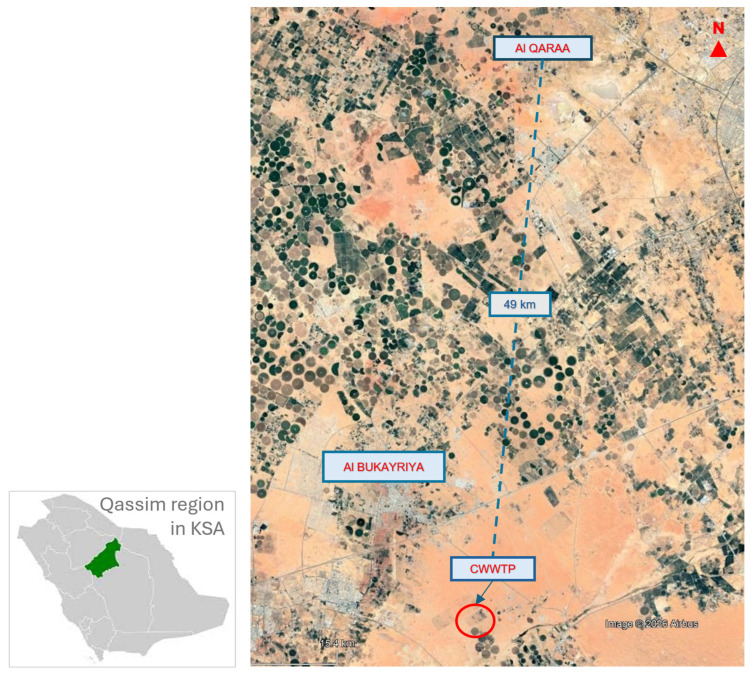
Study region showing the location of Al Qaraa community and its distance from Al-Bukayriyah centralized wastewater treatment facility in Qassim, Saudi Arabia.

The study region shown in Figure 1 is the small community of Al Qaraa in the Qassim Region of Saudi Arabia. The community located ~30 km East of Buraydah, the capital of Qassim, comprises 160 households and accommodates ~1300 persons. In the absence of a community sanitary drainage system, wastewater from each household is initially collected in a septic tank and subsequently transported by private trucks to the nearest centralized wastewater treatment plant (CWWTP), located 49 km away in Al-Bukayriyah City. The entire process poses various environmental and socio-economic challenges for residents, including odors, soil contamination from leaking septic tanks, transportation costs, time spent calling and waiting for privately owned collection vehicles, and noise from sewage pumping from septic tanks to the transport vehicle.

### 2.2. Wastewater Management in the Study Area

#### 2.2.1. Base Case Scenario—Centralized Wastewater Treatment

**Figure 2:**
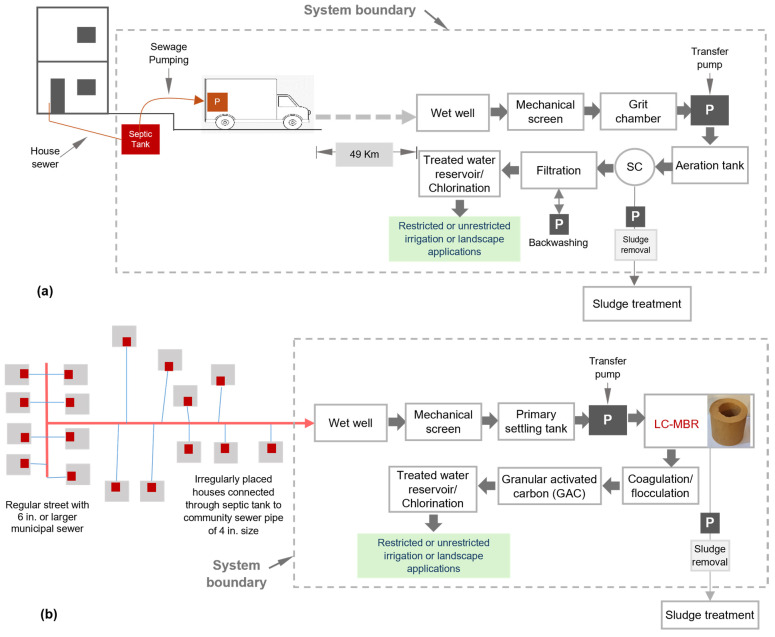
System boundary, (**a**) base case scenario—sewage transportation to a centralized extended aeration activated sludge process (EA-ASP), (**b**) On-site treatment through the decentralized low-cost ceramic filter bioreactor (LC-MBR).

Figure 2a presents the base case scenario and the process flow for the CWWT plant in Bukayriyah. The CWWT facility, with a design capacity of 150,000 m^3^/day, serves a population of around 667,000. Transport vehicles discharge the collected wastewater from the small community’s residences into the wet well. Preliminary treatment consists of mechanical bar screens to remove large and floating objects; subsequently, inorganic suspended solids settle in the grit chamber. Secondary treatment consists of a conventional extended aeration process, comprising an aeration tank and secondary clarifiers, for organic matter removal. Tertiary treatment involves rapid sand filtration (RSF) to further polish the secondary clarifier effluent, ensuring it is suitable for irrigation applications ranging from sprinkler irrigation for urban landscapes to drip and overland-flow irrigation for both restricted and unrestricted agriculture. Clarified effluent from RSF is stored in a treated water reservoir, where it is chlorinated to protect the health of agricultural workers. At present, sludge produced by the EA-ASP is conveyed to an off-site sludge treatment facility, where it is processed in sludge drying beds to convert it into a land conditioner.

Table 1 outlines water quality monitoring results, including means and standard deviations, for raw wastewater collected from septic tanks in the study region, EA-ASP’s effluent (from the nearest centralized wastewater treatment plant in Al Bukayriya city), LC-MBR’s effluent, and KSA’s standards for unrestricted irrigation. The table also gives the operating conditions of the EA-ASP and LC-MBR, including flow rate, hydraulic retention time (HRT), organic loading rate (OLR), and sludge retention time (SRT). Table 1 shows that the effluents from both treatment scenarios comply effectively with the regulatory treated effluent standards in KSA.

#### 2.2.2. Alternative Scenario—Decentralized Wastewater Treatment

Figure 2b presents an alternative scenario of a decentralized LC-MBR for wastewater treatment. Authors’ past studies evaluated the feasibility of an LC-MBR as a DWWT facility in the Al Qaraa area. Considering the average water consumption of 250 L per capita per day (lpcd) and a sewage conversion factor of 0.8, the calculated design capacity of the decentralized plant is 260 m^3^/day. The system boundary encompasses the processes that occur after wastewater is collected from a small community sewer and conveyed to the CWWT facility’s wet well. The preliminary treatment, consisting of a bar screen and a primary settling tank, is warranted by the marginal presence of grit in the small sewerage network and by arid environmental conditions with low annual rainfall in the study area.

Secondary treatment replaces the extended aeration process with an LC-MBR, consisting of a cylindrical ceramic membrane module (pore size: 1–5 µm) fabricated from locally available clay and rice husk ash. The system integrates the activated sludge process with aeration and membrane filtration in a single tank [9,22]. The LC-MBR offers several advantages over the conventional activated sludge process, including lower membrane costs, a smaller footprint, longer sludge retention time (reducing sludge waste), and higher treatment efficiency. To avoid odor and vector issues, sludge from the LC-MBR is assumed to be transported to the shared facility with the EA-ASP, given the limitations of sludge handling and processing in small communities.

The NH_4_^+^ concentration in the effluent of the LC-MBR (0.01 mg/L) is lower than that of the EA-ASP (0.10 mg/L) due to the final polishing step, which involves coagulation–flocculation followed by granular activated carbon (GAC) adsorption. The higher average pH of 7.8 in the LC-MBR effluent than 7 in the EA-ASP effluent can be attributed to the GAC matrix, which contains alkaline surface functional groups and mineral ash, and can adsorb acidic species, thereby increasing the pH.

Tertiary treatment is used to enhance nutrient removal, with coagulation–flocculation (CAF) followed by a GAC column. During coagulation–flocculation, ferric chloride (FeCl_3_, LOBA CHEMIE, Mumbai, India) is dosed at 50 mg/L, and the treated water is passed through a GACC with an empty-bed contact time (EBCT) of 30 min. The final disinfection step is chlorination, identical to that used in the base case scenario. Details of LCCF manufacturing are provided in Shafiquzzaman et al.’s study [23], applications of the LC-MBR in Alresheedi et al.’s [9], and the enhanced nutrient removal process using CAF and GAC in Shafiquzzaman et al.’s [22].

### 2.3. Life Cycle Assessment Methodology

The present study used the following LCA principles established by the International Organization for Standardization (ISO): Environmental management—Life cycle assessment—Principles and framework (ISO 14040:2006) [24] and Environmental management—Life cycle assessment—Requirements and guidelines (ISO 14044:2006) [25].

#### 2.3.1. Goal and Scope

The present study aims to investigate the environmental impacts of an LC-MBR-type decentralized wastewater treatment plant for smaller communities in Saudi Arabia, and to compare them with the existing centralized treatment scenario of the EA-ASP. The goal of the treatment scenarios is to ensure compliance with regulatory standards for unrestricted irrigation reuse of treated wastewater. A cradle-to-gate approach was adopted, excluding the end-of-life or decommissioning phase due to its negligible contribution to the LCA of wastewater treatment [23].

The data for the base case (transportation + effluent from a CWWT plant) scenario were obtained from the National Water Company (NWC) operating in Buraydah, Qassim, Saudi Arabia, and from Alresheedi et al. [9] for the LC-MBR-based decentralized scenario.

#### 2.3.2. Functional Unit

Past studies have used 1 m^3^ [26,27] or person equivalents (PE) [28] as functional units for LCA of water and wastewater facilities. The present study uses a 1 m^3^ functional unit for treated effluent and a lifespan of 50 years, as is commonly used in wastewater treatment plant design. The centralized and decentralized WWT plants aim to meet the unrestricted irrigation COD standard of less than 50 mg/L in Saudi Arabia [29]. The study assumes that the decentralized LC-MBR facility can be expanded incrementally by adding standardized units as population grows, without major modifications to existing infrastructure or changes in process efficiency.

#### 2.3.3. System Boundary

**Figure 3:**
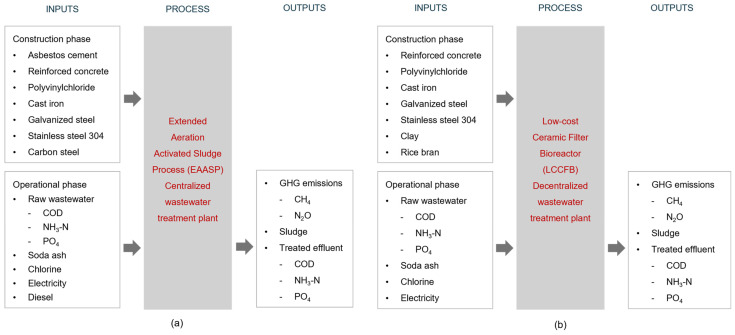
System boundaries showing inputs, process, and outputs for centralized and decentralized wastewater treatment scenarios; (**a**) extended aeration activated sludge process (EA-ASP); (**b**) low-cost ceramic filter bioreactor (LC-MBR).

Figure 3 illustrates the system boundaries and the inputs and outputs of the centralized EA-ASP and decentralized LC-MBR processes. The system boundaries include both the construction and operational phases of the treatment scenarios. The construction phase encompasses all materials used in primary, secondary, and tertiary treatment processes. The operational phase includes COD, ammonium nitrogen (NH_4_^+^-N), and orthophosphate (PO_4_^3−^) as the most critical water quality parameters for health and ecological impacts. Transportation of materials was excluded from the system boundary because the supply chains, transport distances, vehicle types, and fuel use were identical across the two scenarios, yielding nearly equivalent transportation-related environmental impacts.

Presently, the sludge produced from the EA-ASP is transported to a sludge treatment facility located away from the plant site. The sludge from the decentralized LC-MBR is assumed to be conveyed to the same facility due to technical limitations and potential odor and vector-related issues, thereby avoiding the complexities of sludge handling and processing in smaller communities. In addition to the fact that sludge is a co-product rather than the main functional output, the present study excluded sludge treatment from the LCA by ignoring differences in sludge volume produced by the two WWT scenarios, the unavailability of sludge management data, and the assumption of the same potential environmental impacts in both scenarios.

CH_4_ and N_2_O generation from wastewater treatment plants was considered the primary contributor to greenhouse gas emissions. CO_2_ was included in air emissions because of its biogenic origin, leading to its interpretation as climate-neutral [30]. The operational phase of the base case scenario includes electricity for pumping and fuel (diesel) for transporting wastewater from a small community to the CWWT facility.

#### 2.3.4. Inventory Analysis for Centralized Facility

The required construction materials for the EA-ASP, reported as kg/m^3^, were obtained from the literature [17]. Electricity consumption for primary, secondary, and tertiary treatment of the EA-ASP in Al Bukayriya City was obtained from the NWC office in Buraydah City: 3.35 × 10^−1^ kWh/m^3^ for primary (coarse mechanical screens and grit pumps), 2.01 × 10^−1^ kWh/m^3^ for secondary (aerators, bridge motors, and screw pumps), and 6.7 × 10^−2^ kWh/m^3^ for tertiary (RSF, chlorine gas dosing system, chemical pumps, decanters, and sludge conveyor motors). The total energy consumption of 0.6 kWh/m^3^ at the centralized facility is within the reported range of 0.3 to 1.12 kWh/m^3^ for the EA-ASP [31,32]. A fossil-fuel-driven electricity grid supplies energy to the Al Qaraa area. One of the primary differences between centralized and decentralized wastewater treatment options in the present study is the diesel required to transport household wastewater to the centralized treatment facility every 21 days. One thousand three hundred (1300) persons residing in 160 households, with an average water consumption of 250 lpcd, generate 260 m^3^/day of wastewater.

Centralized treatment facilities in Qassim are designed to produce effluent that meets unrestricted irrigation standards, as follows: 5 mg/L NH_4_^+^-N; 10 mg/L BOD_5_; 50 mg/L COD; 10 mg/L TSS; and 10 mg/L PO_4_^3−^ (Table 1). One-year effluent data obtained from the EA-ASP in Al-Bukayriyah city showed reliable performance with mean values of 0.1 mg/L (NH_4_^+^-N), 5 mg/L (BOD_5_), 18.5 mg/L (COD), 2.1 mg/L (TSS), and 7.5 mg/L (PO_4_^3−^). Septic tanks designed for 21 days of storage contain 5460 m^3^ of domestic wastewater for subsequent transfer to the CWWT facility. Background concentrations of air emissions, CH_4_ (8.0 × 10^−3^) and N_2_O (3.38 × 10^−3^) generated from EA-ASP operations, were obtained from Campos et al. [33]. Trucks of carrying capacity of 32 m^3^, covering a 49 km distance from Al Qaraa to the Al-Bukayriyah CWWT facility, consume 2.87 × 10^−4^ m^3^ of diesel per m^3^ of wastewater. Detailed inventory data for EA-ASP are provided in Appendix A.

#### 2.3.5. Inventory Analysis for Decentralized Facility

For the on-site decentralized LC-MBR scenario, material quantities for the construction of primary treatment facilities (wet well, screens, and grit chamber), secondary (aeration tank), and tertiary (coagulation and GAC units) facilities, such as cast iron, mild steel, stainless steel, and reinforced concrete, were obtained from the literature on system type [17]. Detailed inventory data for the EA-ASP are provided in Appendix B.

The amounts of clay and rice bran required to manufacture a low-cost ceramic membrane with a two-year lifespan were estimated at 15 kg and 4 kg per m^3^ of wastewater, respectively. Electricity consumption of 1.09 × 10^−1^ kWh/m^3^ for primary, 2.2 × 10^−1^ kWh/m^3^ for secondary, and 3.27 × 10^−1^ kWh/m^3^ for tertiary treatment was estimated during the operational phase, based on the authors’ bench-scale LC-MBR [9]. The total energy consumption of 0.656 kWh/m^3^ is comparable to the reported values for simplified wastewater treatment processes, such as 1.07 kWh/m^3^ for the hollow-fiber membrane bioreactor (MBR) [34], 0.19 kWh/m^3^ for the anaerobic MBR [35], and 1.042 kWh/m^3^ for a sequential batch reactor [12]. The effluent quality data for BOD_5_ (4.6 mg/L), COD (18.1 mg/L), and TSS (0.9 mg/L) were taken from Alresheedi et al. [9] for the LC-MBR. Authors in a recent study [22] achieved NH_4_^+^-N (0.85 mg/L) and PO_4_^3−^ (4 mg/L) by applying coagulation and GAC after an LC-MBR. In the absence of actual plant data, background concentrations of CH_4_ (7.5 × 10^−3^) and N_2_O (3 × 10^−3^) generated by the LC-MBR were assumed based on a previous study [33]. All the parameters meet KSA standards for unrestricted irrigation (Table 1).

### 2.4. Impact Assessment

LCA was performed using SimaPro 8.3.0.0, and the ecoinvent database version 3.0 was used for background processes. The allocation method is mass allocation because environmental impacts are distributed in proportion to each product’s quantity. The study adopted hierarchical ReCiPe 16 midpoint and endpoint methodologies for impact assessment. The study considered a set of fifteen relevant midpoint impact categories, comprising climate change, terrestrial acidification, freshwater eutrophication, marine eutrophication, human toxicity, photochemical oxidant formation, particulate matter formation, freshwater ecotoxicity, marine ecotoxicity, agricultural land occupation, urban land occupation, natural land transformation, water depletion, metal depletion, and fossil depletion, together with endpoint categories addressing human health, ecosystems, and resources. The midpoint categories were selected based on the following reasons: (i) extensive fossil fuel consumption for energy production [36]; (ii) water pollutants and air emissions from wastewater treatment processes [33]; (iii) dry and low-flow freshwater bodies that offer small dilution to wastewater discharges [1]; (iv) effluent discharges into the marine environment for areas near coasts [37]; and (v) impact of raw material used for the LC-MBR on land use and terrestrial environment.

## 3. Results

### 3.1. Impact Assessment for Centralized and Decentralized Facilities

**Figure 4:**
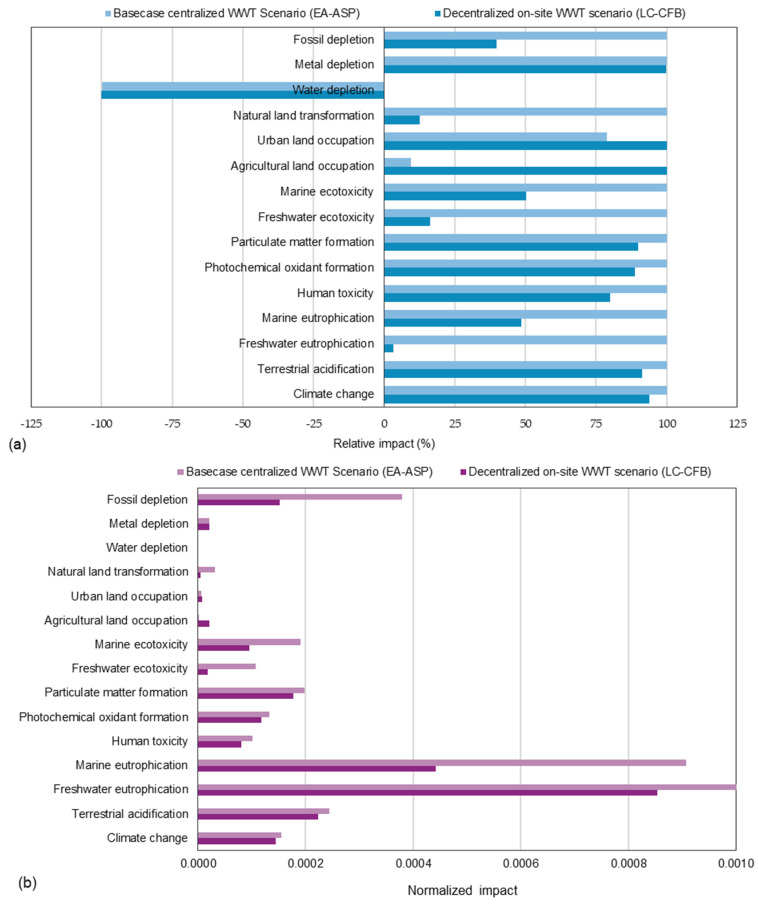
LCA results for base-case CWWT (EA-ASP) and on-site DWWT (LC-MBR) scenarios for smaller communities in arid regions: (**a**) relative impacts; (**b**) normalized impacts.

The characterization of 15 selected categories in Table 2 shows overall higher impacts for the EA-ASP. Figure 4a demonstrates the relative impacts across 15 categories chosen for the centralized base case WWT using the EA-ASP and the decentralized on-site WWT using the LC-MBR. The decentralized option clearly outperforms the centralized option in reducing freshwater eutrophication, with only 3.3% relative impact, owing to the removal of nearly half of the phosphorus concentration through coagulation and GAC (Table 1). For the same reason, freshwater ecotoxicity is the second category in which the LC-MBR shows a 6-fold lower impact than the EA-ASP. The decentralized scenario also accounts for nearly half of the effects in the categories of fossil depletion (~40%) and marine eutrophication (~49%). Both WWT scenarios performed similarly across categories assessing particulate matter and photochemical oxidant formation, with the decentralized WWT scenario exhibiting around 10% lower impacts. The EA-ASP outperforms the decentralized scenario in agricultural land occupation (~9%) and urban land occupation (~79%), driven by clay and rice bran in LC-MBR manufacturing. The LC-MBR also showed relatively lower impacts on photochemical oxidant formation (~87%) and particulate matter formation (~90%) than the EA-ASP, mainly because it omitted wastewater transportation. In the metal depletion and climate change scenarios, both showed significant implications due to the use of fossil fuels for electricity generation.

Figure 4b shows the normalized environmental impact for all categories, obtained using the ReCiPe 16 midpoint world reference method. Freshwater eutrophication (2.61 × 10^−2^) and marine eutrophication (9.07 × 10^−4^) were found to be the highest environmental impacts by the EA-ASP. The findings align with Chen et al. [38] on the environmental impact assessment of effluent from a full-scale WWTP, highlighting climate change and eutrophication as the main contributors to these impacts. Fossil depletion ranked third among the EA-ASP’s environmental impacts, with a characterized impact value of 3.80 × 10^−4^. In all three categories, the LC-MBR exhibits normalized environmental impacts that are more than 50% lower than those of the base case scenario. These findings are consistent with expectations, as climate change impacts are fundamentally associated with fossil fuel depletion, especially when operational energy relies on them [39].

The centralized scenario of the EA-ASP exhibits relatively low normalized impacts in the climate change (1.54 × 10^−4^), photochemical oxidant formation (1.32 × 10^−4^), and particulate matter formation (1.98 × 10^−4^) categories. Nevertheless, the decentralized LC-MBR scenario exhibits approximately 6–11% lower impacts across these categories. Normalization showed approximately 20% lower human toxicity impact for the LC-MBR (8.06 × 10^−5^) than for the EA-ASP (1.01 × 10^−4^). Likewise, the EA-ASP exhibits a freshwater ecotoxicity impact that is over 80% higher (1.07 × 10−4) than that of the LC-MBR (1.73 × 10^−5^). Although the normalized values were very small overall, the use of raw materials (clay and rice bran) for membrane manufacturing resulted in approximately 91% higher impacts on agricultural land occupation and about 21% higher impacts on urban land occupation compared to the EA-ASP system. The analysis showed minimal normalized impacts for metal depletion, and a positive impact of treated effluent on water depletion was observed across both scenarios.

### 3.2. LCA Contribution for Wastewater Treatment Scenarios

#### 3.2.1. Base Case Centralized Wastewater Treatment (EA-ASP)

**Figure 5:**
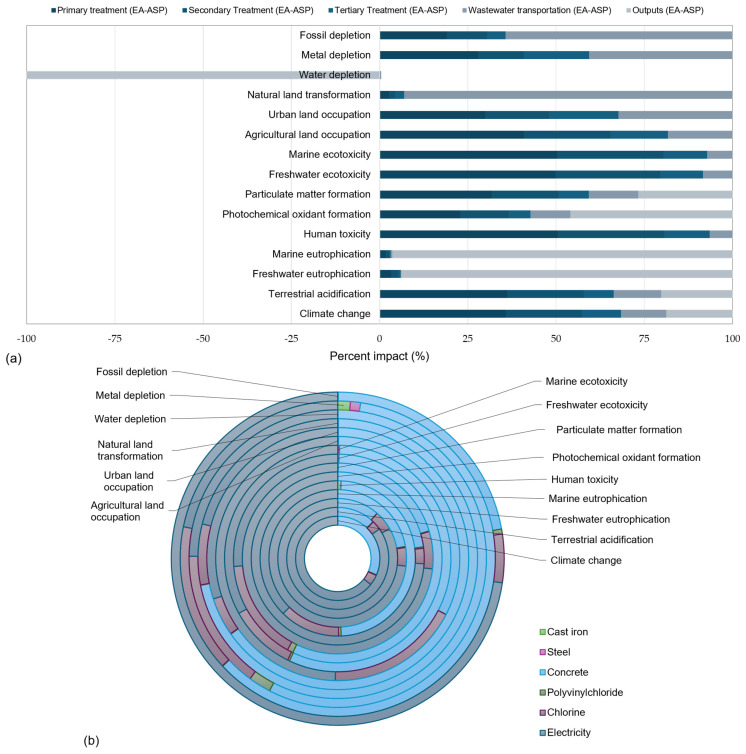
LCA results for base case centralized wastewater treatment scenario (EA-ASP), (**a**) environmental impact percentage contribution of different treatment stages, (**b**) contribution to significant midpoint categories by tertiary treatment.

Figure 5a illustrates the percentage relative contribution of each treatment process in the CWWT scenario. Primary treatment with the largest electricity consumption by wastewater pumping has the most substantial impacts on climate change (~36%), terrestrial acidification (~36%), human toxicity (~50%), particulate matter formation (~32%), freshwater ecotoxicity (~50%), and agricultural land occupation (~41%). Secondary treatment mainly contributes to human toxicity (~30%), freshwater ecotoxicity (~30%), and marine ecotoxicity (~30%). The most significant impacts from wastewater transportation were natural land transformation (93%) and fossil depletion (~64%). The most critical impact categories from wastewater discharges were marine eutrophication (~96%), freshwater eutrophication (~94%), and photochemical oxidant formation (~46%). High eutrophication impacts can be associated with the remaining orthophosphate in the treated effluent. The most significant positive impact (−100%) of wastewater output was on water depletion, owing to the strong potential to treat effluent for reuse.

Figure 5b presents the contributions of different materials and processes to environmental impact categories during tertiary treatment. Electricity and concrete made the most significant contributions to overall impacts across almost all categories. Chlorine registered noticeable contributions to freshwater ecotoxicity (~16%), agricultural land occupation (~18%), water depletion (~15%), and metal depletion (~15%), attributing to its aquatic toxicity potential for freshwater species—such as fish, crustaceans, and insects [40]—raw material use for chlorine production [41], water requirements for processing and cooling [42], and use of metals and mineral, e.g., electrodes and catalysts, during production [43].

#### 3.2.2. On-Site Decentralized Wastewater Treatment (LC-MBR)

**Figure 6:**
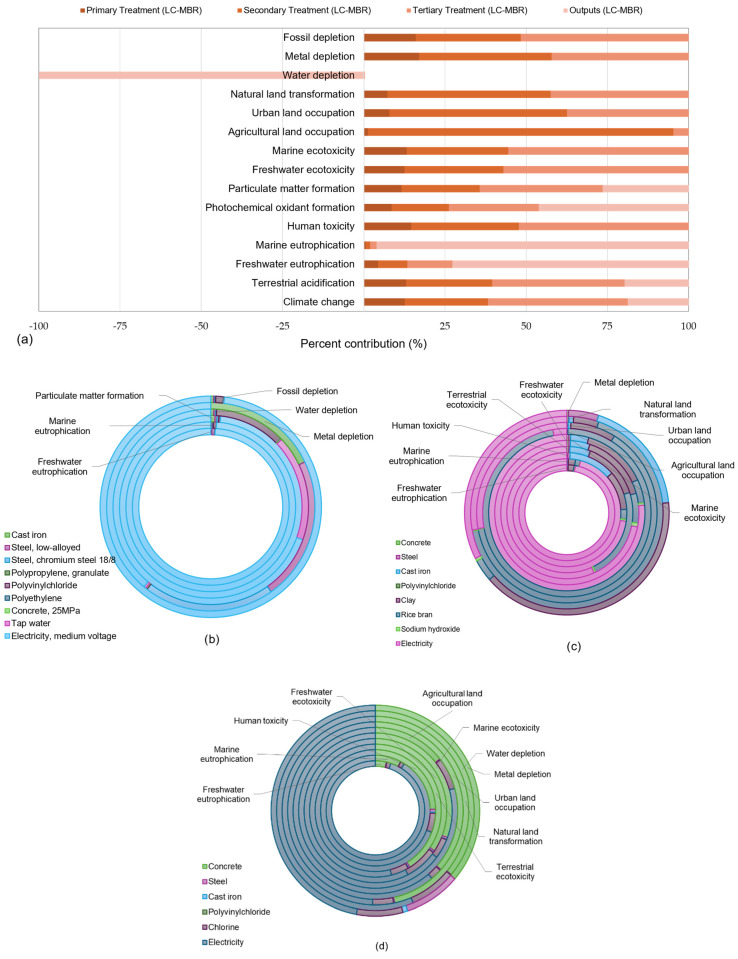
LCA results for on-site decentralized wastewater treatment scenario (LC-MBR); (**a**) environmental impact percentage contribution of different treatment stages; (**b**) contribution of materials and processes by primary treatment; (**c**) secondary treatment; and (**d**) tertiary treatment.

Figure 6a shows the relative contributions of each treatment process in the decentralized LC-MBR scenario. Metal depletion (~17%), fossil depletion (~16%), and human toxicity (~15%) are the main categories impacted by the primary treatment processes. Secondary treatment primarily contributed to metal depletion (~41%), fossil depletion (~32%), human toxicity (~33%), freshwater ecotoxicity (~30%), and marine ecotoxicity (~31%). In the base case scenario, the secondary clarifier employed materials and manufacturing processes that somehow traded off against installing a low-cost membrane in the aeration tank, impacting agricultural land occupation (~94%), urban land occupation (~55%), and natural land transformation (~50%). From tertiary treatment, the most significant impacts were freshwater ecotoxicity (57%), marine ecotoxicity (~55%), human toxicity (~52%), fossil depletion (~52%), climate change (~43%), metal depletion (~42%), and particulate matter formation (~38%). Similar to the CWWT scenario, the top impact categories from wastewater outputs were marine eutrophication (~96%), freshwater eutrophication (~73%), and photochemical oxidant formation (~46%), potentially associated with residual nutrients and emissions from the treated effluent. The wastewater effluent had the greatest negative impact (–100%) on water depletion, indicating its high potential for reuse.

Figure 6b–d illustrate the percentage contributions of processes and materials to different impact categories for primary, secondary, and tertiary treatment in the decentralized LC-MBR scenario. Electricity, tap water, cast iron, steel, and Polyvinylchloride are the largest contributors from primary treatment. Furthermore, clay and rice bran for membrane manufacturing were added in the secondary treatment process. Concrete and electricity were identified as the primary contributors to tertiary treatment.

### 3.3. Endpoint Impact Categories

**Figure 7:**
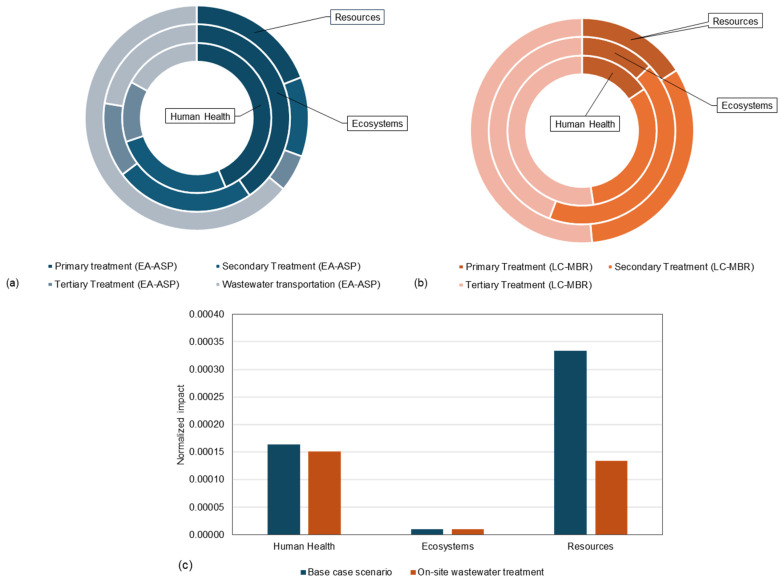
LCA results showing relative impacts of different types of treatment on endpoint impact categories, (**a**) base case centralized wastewater treatment scenario (EA-ASP), (**b**) on-site decentralized wastewater treatment scenario (LC-MBR), (**c**) normalized impacts.

Figure 7a presents LCA results for the CWWT scenario (EA-ASP), demonstrating the impact of different treatment levels and outputs on endpoint categories, including human health, ecosystems, and resources. Primary treatment poses the greatest threat to human health and ecosystems due to high energy consumption for wastewater pumping. In terms of energy consumption, the analysis revealed that primary treatment had approximately 40% higher impacts than secondary treatment and more than 70% higher impacts than tertiary treatment across all three categories. Wastewater transportation had a 70% higher impact on resources than primary treatment, 82% than secondary treatment, and 92% than tertiary treatment, due to the use of diesel for vehicles.

The LCA results in Figure 7b for the DWWT scenario (LC-MBR) showed ~70% higher impacts from the tertiary treatment process than from primary treatment, due to the use of several pumps across multiple treatment processes for nutrient removal (Figure 2). Tertiary treatment also has around 40% higher impacts on human health and resources than secondary treatment. However, the impact on the ecosystem is almost the same, as local materials are used in membrane manufacturing. Normalized endpoint impacts in Figure 7c clearly show that the LC-MBR has a significantly lower impact on resources, attributable to avoiding wastewater transport and lower operational energy requirements.

## 4. Discussion

LCA highlights the following normalized impacts for the WWT scenarios as the top six impact categories: freshwater eutrophication (EA-ASP: 2.61 × 10^−2^ and LC-MBR: 8.54 × 10^−4^), marine eutrophication (EA-ASP: 9.07 × 10^−4^ and LC-MBR: 4.42 × 10^−4^), fossil depletion (EA-ASP: 3.80 × 10^−4^ and LC-MBR, 1.51 × 10^−4^), particulate matter formation (EA-ASP: 1.98 × 10^−4^ and LC-MBR: 1.78 × 10^−4^), climate change (EA-ASP: 1.54 × 10^−4^ and LC-MBR: 1.45 × 10^−4^), and photochemical oxidant formation (EA-ASP, 1.32 × 10^−4^ and LC-MBR, 1.17 × 10^−4^). Human toxicity (EA-ASP: 1.01 × 10^−4^ and LC-MBR: 8.06 × 10^−5^), freshwater ecotoxicity (EA-ASP: 1.07 × 10^−4^; LC-MBR: 1.73 × 10^−5^), and metal depletion (EA-ASP, 2.09 × 10^−5^; LC-MBR, 2.09 × 10^−5^) were also identified as essential impact categories in the overall environmental burden. On-site DWWT using the LC-MBR shows a lesser life cycle environmental impact in all categories compared to the conventional, more complex centralized EA-ASP, which complies with the findings of past studies on low-cost technologies with simplified operations, such as sequential batch reactors [12,17], slow rate filtration plants [19], and constructed wetlands [18].

Electricity generated from fossil fuels accounts for the largest share across all midpoint categories in the centralized (EA-ASP) case, owing to the use of four pumps for the bar screens and the grit chamber. The total energy consumption of 0.6 kWh/m^3^ for the EA-ASP, falling almost in the middle of the reported range of 0.3–1.12 kWh/m3 [29,30], can be attributed to efficient aeration, which is the most energy-intensive process in an ASP. Controlling dissolved oxygen levels through optimal aeration durations can directly reduce blower energy demand. Better oxygen-transfer efficiency, affected by diffuser type, diffuser fouling, bubble size, and the depth of the aeration tank, also minimizes energy per unit of oxygen delivered [44]. A higher BOD/COD ratio, reflecting a larger proportion of biodegradable organic matter, also minimizes oxygen requirements, or, in other words, energy requirements [45]. Such higher ratios can be attributed to the promulgation of strict regulations requiring permits from the National Center for Environmental Compliance (NCEC) for industrial discharges into community sewers conveying wastewater to the CWWT [46]. Higher temperatures in the study area also enhance biological activity and oxygen-transfer efficiency, thereby reducing aeration requirements [44].

In contrast, the contributions of cast iron, steel, and PVC are also significant in the water depletion and metal depletion categories, owing to the use of one pump each for bar screens and the primary sedimentation tank in the decentralized (LC-MBR) system. These findings align with the study by Mamathoni and Harding [17] on the EA-ASP and a small-scale sequential batch reactor in South Africa. The primary contributor (>95%) is electricity generated from fossil fuels for the following categories: freshwater and marine eutrophication (~99%), particulate matter formation (98.2%), and fossil depletion (~98%). For the water depletion impact category, 69.5% of the contribution is from electricity and 11.8% from Polyvinyl Chloride (PVC), due to extensive water use during polymerization and resin production. In polymerization, water is used both for cooling and, indirectly, for energy generation and chemical processing. A study by Olapiriyakul [47] in Thailand reported that 14.72 L of water is used to produce 1 Kg of resin. Figure 6b also shows contributions from low-alloy steel (22.3%), chromium steel (20.3%), and cast iron (17.9%) to metal depletion. Low-alloy steel is used to manufacture bars, structural frames, and rake arms of screens, as well as the shafts and impellers of sewage pumps, owing to its higher tensile strength and fatigue resistance. Cast iron and chromium steel are used for pump casings, impellers, and volutes due to their good wear resistance and damping characteristics [48].

In secondary treatment, the contribution from the construction phase of the EA-ASP to the overall environmental impacts remained less than 1%, and operational electricity for aeration and pumping was the primary contributor (~99%) across all impact categories. In the case of the LC-MBR, the following contribution of clay and rice bran was found on different impact categories: human toxicity (13.3%), terrestrial ecotoxicity (37%), freshwater ecotoxicity (22.1%), marine ecotoxicity (18.4%), agricultural land occupation (97.3%), urban land occupation (71.3%), and natural land transformation (70.8%). The use of clay and rice bran in the secondary treatment process of the LC-MBR contributed to human toxicity, terrestrial, freshwater, and marine ecotoxicity, agricultural land occupation, urban land occupation, natural land transformation, and metal depletion. Although some of these categories do not contribute significantly to the overall LCA results, the impacts of different processes and materials are briefly outlined to acknowledge the use of a low-cost membrane in the secondary treatment stage. Human exposure to clay may pose a risk of lung damage [49]. LCA shows that clay contributes 10.6% to human toxicity, particularly due to its silica content. Rice bran indirectly influences terrestrial ecotoxicity, contributing 22.8% through effects on nutrient recycling and microbial balance [50]. Clay’s contribution of 15.5% to freshwater ecotoxicity and 13.5% to marine ecotoxicity can be attributed to mining effluents and chemical use during extraction and washing [51]. A large share of rice bran was observed in the agricultural (97.1%) and urban (63.2%) land occupation impact categories, driven by the need for land for biomass production and manufacturing infrastructure. Electricity remained the primary contributor to several categories: freshwater and marine eutrophication (~95%), human toxicity (72.7%), terrestrial ecotoxicity (56.7%), freshwater ecotoxicity (71.8%), and marine ecotoxicity (76.6%).

After electricity, concrete manufacturing is a significant contributor to almost all impact categories in the tertiary treatment phase of the EA-ASP, except for freshwater and marine eutrophication. Approximately 11.4% of the freshwater eutrophication burden is attributable to concrete, 5.3% to chlorine, and ~83% to electricity consumption. Likewise, electricity, with approximately 73% of the contribution, remained at the top, affecting marine eutrophication, particulate matter formation, and fossil depletion. The contributions of concrete and chlorine to these impact categories are 20.2–22.2% and 4.4–6.3%, respectively. Polyvinyl chloride accounts for about 5% of fossil fuel depletion. High electricity demands for wastewater pumping and aeration at the primary and secondary treatment levels impose an environmental burden exceeding 90% across all three impact categories.

The on-site DWWT scenario using the LC-MBR offers a practical solution for rural settings in KSA. The LC-MBR is a technically and economically viable approach to addressing key wastewater management challenges in the Kingdom, particularly under conditions of severe water scarcity, rising wastewater generation, and the national objective of maximizing water reuse. Technology provides an opportunity to promote decentralized, low-energy, and resource-efficient wastewater treatment as an alternative to the substantial reliance on desalination and the high energy footprint of centralized wastewater treatment systems in KSA. Moreover, the LC-MBR enables the reuse of treated effluent for landscaping and irrigation in local communities, thereby reducing dependence on freshwater abstraction and desalinated water.

In arid regions, including Saudi Arabia, the LC-MBR is particularly appropriate due to its low membrane production costs and the potential to manufacture membranes from locally available raw materials such as clay and agricultural residues, reducing reliance on imported polymeric membranes and aligning with national localization and industrial development initiatives under Saudi Vision 2030 [52]. The lower capital cost makes the LC-MBR especially suitable for small and remote communities, as well as tourist facilities, where conventional centralized infrastructure is either unavailable or cost-prohibitive. From an operational perspective, the LC-MBR offers advantages in compact footprint, process integration, and operational simplicity, all of which are critical in arid environments. The integration of aeration, biological treatment, and solid–liquid separation into a single unit, thereby reducing infrastructure requirements and enabling modular, scalable deployment, is particularly relevant for decentralized wastewater treatment in rapidly growing peri-urban areas and remote settlements.

Although economic analysis or life-cycle costs were beyond the scope of the present study, previous studies have reported lower operational costs for ceramic-based membrane bioreactors than for conventional membrane systems and activated sludge processes. Sun and Jin [53] reported 62.5% lower energy costs for a ceramic membrane bioreactor than for the conventional MBR process in a pilot-scale comparative study in Japan. Even when not manufactured from low-cost local materials, ceramic membranes—despite higher upfront material costs than polymeric membranes—can operate efficiently over longer periods and withstand thermal variations, resulting in lower life-cycle costs [54]. Generally, MBR systems produce less sludge than conventional ASP systems because they operate at higher solids concentrations, thereby reducing sludge handling, transportation, and disposal costs [12].

Overall, the LC-MBR is a promising technology for advancing sustainable wastewater management in Saudi Arabia, offering a low-cost, decentralized, and energy-efficient alternative to conventional treatment systems. Its adoption could enhance water reuse, reduce environmental impacts, and support national sustainability and localization objectives, particularly for small-scale and decentralized applications. With adequate policy support, pilot-scale deployment, and capacity-building initiatives, LC-MBR systems have the potential to play a central role in Saudi Arabia’s decentralized wastewater treatment strategy and in advancing sustainable water and resource management.

Some smaller communities in Saudi Arabia are facing various socio-economic and environmental challenges related to wastewater management due to the absence of sewerage networks and on-site treatment facilities. Clean water and sanitation and sustainable cities and communities are two of the seventeen United Nations Sustainable Development Goals (SDGs) [41,49]. In support of the United Nations Educational, Scientific, and Cultural Organization (UNESCO), the Government of KSA developed and implemented Vision 2030 for sustainable development, with waste recycling, pollution control, and resource conservation among its primary objectives [55,56].

Al-Miyah Solutions, in collaboration with KAUST Innovation and NWC, developed a mobile, decentralized WWT system capable of treating 150 m^3^ of wastewater per day, comprising aerobic granular sludge to enhance microorganism size, a gravity-driven membrane bioreactor, and an ultraviolet disinfection unit [57]. Authors have also developed a bench-scale LC-MBR for decentralized WWT in smaller communities [7]. A later study evaluating willingness to accept, based on consumers’ perceptions of the LC-MBR technology and its associated benefits, reveals a moderately high willingness to reuse [3]. The study also highlighted the need to enhance education on sustainable development and resource conservation for the long-term sustainability of DWWT options through public–private partnerships in smaller communities.

## 5. Conclusions

Smaller communities in KSA store wastewater in septic tanks and then transfer it to the nearest centralized facility, which is often located far away. The concerned agencies are seeking sustainable, decentralized wastewater treatment options to address the socio-economic (costs and nuisances associated with wastewater pumping and transportation) and resources (inability to reuse the treated effluent) concerns of these communities. The present study conducted an LCA (based on 1 m^3^ of treated effluent) evaluation of an extended-aeration-activated-sludge-process-type centralized WWT plant located 49 km from the study region and a low-cost membrane bioreactor-type decentralized facility. Considering relative and normalized impacts, the decentralized scenario outperforms the centralized option in 13 of 15 impact categories. Owing to high energy requirements, primary treatment in a centralized EA-ASP had the greatest impact across all categories, except for freshwater and marine eutrophication. Secondary treatment in the decentralized LC-MBR scenario had the greatest relative impact on urban and agricultural land-use categories, driven by the use of clay and rice bran for low-cost membrane manufacturing.

Nevertheless, the normalized impacts of these categories were manyfold lower than those of other categories; for instance, 2.09 × 10^−5^ for the LC-MBR compared to 1.96 × 10^−6^ for the EA-ASP under agricultural land occupation. Tertiary treatment in the LC-MBR scenario, through coagulation and GAC, minimized freshwater eutrophication, resulting in a 3.3% relative impact and a phosphorus concentration nearly half that of the EA-ASP’s effluent. Therefore, tertiary treatment significantly contributes to climate change, human toxicity, particulate matter formation, marine ecotoxicity, and fossil depletion, driven by concrete use during construction and fossil-fuel-based electricity during operation. Based on normalized endpoint impacts, the LC-MBR showed 8% lower impact on human health and 60% lower impact on resources, while both scenarios showed similar (though insignificant) impacts on the ecosystem. The present study helps policy and decision-makers evaluate the sustainability of low-cost, decentralized wastewater treatment systems for smaller communities in arid regions.

Future studies can examine the impacts of sludge management processes in comparative LCA analyses of decentralized wastewater treatment scenarios. A pilot-scale study is also recommended to evaluate the practicality of the LC-MBR for decentralized wastewater treatment in smaller communities in arid regions. Real-time monitoring of gaseous emissions from pilot-scale application will improve the accuracy of future LCA studies on low-cost membrane bioreactors. Further studies could conduct economic analyses to compare the life-cycle costs of LC-MBR systems with those of other membrane-based technologies or conventional activated sludge processes.

## Figures and Tables

**Table 1:** Operating conditions and water quality monitoring results of the extended aeration activated sludge process (EA-ASP) and the low-cost ceramic filter bioreactor (LC-MBR).

No.	Parameter	Units	Septic Tank Effluent Concentration [9]	Effluent of EA-ASP (CWWT) [1]	Effluent of LC-MBR (DWWT) [9,22]	KSA Standards for Unrestricted Irrigation [9]
A	Water quality monitoring					
1	pH	-	7.3 ± 0.32	7 ± 0.1	7.8 ± 0.31	6–8.4
2	Biochemical oxygen demand (BOD5)	mg/L	160 ± 12.31	5.0 ± 0.6	4.6 ± 2.8	10
3	Chemical oxygen demand (COD)	mg/L	386 ± 14.62	18.5 ± 1.8	18.1 ± 3.4	50
4	Total suspended solids (TSS)	mg/L	157 ± 22.54	2.1 ± 0.5	0.9 ± 0.05	10
5	Ammonium Nitrogen (NH_4_^+^-N)	mg/L	34 ± 3.55	0.1 ± 0.1	0.1 ± 0.001	5
6	Orthophosphate (PO_4_^3−^)	mg/L	18 ± 2.88	7.5 ± 1.0	4.0 ± 0.24	10
B	Operating conditions					
7	Flow rate	m^3^/d	-	90.0 × 10^3^	2.6 × 10^2^	-
8	Hydraulic retention time (HRT)	h	-	21	36	-
9	Organic loading rate (OLR)	kg COD/m^3^·d	-	0.5	0.15	-
10	Sludge retention time (SRT)	day	-	20	21	-

**Table 2:** LCA characterization for centralized (EA-ASP) and decentralized (LC-MBR) wastewater treatment scenarios.

Impact Category	Units	EA-ASP	LC-MBR
Climate change	kg CO_2_ eq	1.06 × 10^0^	9.98 × 10^−1^
Terrestrial acidification	Kg SO_2_ eq	9.34 × 10^−3^	8.52 × 10^−3^
Freshwater eutrophication	kg P eq	7.56 × 10^−3^	2.47 × 10^−4^
Marine eutrophication	kg N eq	6.70 × 10^−4^	3.25 × 10^−3^
Human toxicity	kg 1,4-DB eq	3.28 × 10^−2^	2.63 × 10^−2^
Photochemical oxidant formation	kg NMVOC	7.52 × 10^−3^	6.67 × 10^−3^
Particulate matter formation	kg PM_10_ eq	2.78 × 10^−3^	2.50 × 10^−3^
Freshwater ecotoxicity	kg 1,4-DB eq	4.70 × 10^−4^	7.47 × 10^−5^
Marine ecotoxicity	kg 1,4-DB eq	4.70 × 10^−4^	2.36 × 10^−4^
Agricultural land occupation	m^2^a	1.06 × 10^−2^	1.14 × 10^−1^
Urban land occupation	m^2^a	4.97 × 10^−3^	6.31 × 10^−3^
Natural land transformation	m^2^	3.75 × 10^−4^	4.67 × 10^−5^
Water depletion	m^3^	−9.96 × 10^−1^	−9.97 × 10^−1^
Metal depletion	kg Fe eq	9.28 × 10^−3^	9.27 × 10^−3^
Fossil depletion	kg oil eq	4.90 × 10^−1^	1.95 × 10^−1^

## Data Availability

The raw data supporting the conclusions of this article will be made available by the authors on request.

## Abbreviations

The following abbreviations are used in this manuscript:

CAF	Coagulation–flocculation
COD	Chemical Oxygen Demand
CWWT	Centralized wastewater treatment
DWWT	Decentralized wastewater treatment
EA-ASP	Extended aeration activated sludge process
EBCT	Empty bed contact time
GACC	Granular activated carbon column
KSA	Kingdom of Saudi Arabia
LCA	Life cycle analysis
LC-MBR	Low-cost ceramic membrane bioreactor
MBR	Membrane bioreactor
RSF	Rapid sand filtration

## Appendix A. Life Cycle Inventory for the Extended Aeration Activated Sludge Process (EA-ASP)

**Inputs**	**Quantity**	**Units**	**Description**	**Ecoinvent 3.6 Market Process**
Primary treatment				
Cast Iron ^a^	8.00 × 10^−4^	kg/m^3^	Cast iron	{GLO}j market for j Cutoff, U
Galvanized Steel ^a^	6.50 × 10^−6^	kg/m^3^	Steel, low-alloyed	{GLO}j market for j Cutoff, U
Reinforced Concrete ^a^	1.60 × 10^−6^	m^3^/m^3^	Concrete, 25 MPa	{GLO}j market for j Cutoff, U
Polyvinylchloride ^a^	3.80 × 10^−6^	kg/m^3^	Polyvinylchloride, emulsion polymerised	{GLO}j market for j Cutoff, U
Electricity ^b^	3.35 × 10^−1^	Kwh/m^3^	Electricity, medium voltage	{ZA}j market for j Cutoff, U
Secondary Treatment				
Carbon Steel ^a^	6.50 × 10^−6^	kg/m^3^	Steel, low-alloyed	{GLO}j market for j Cutoff, U
Stainless Steel 304 ^a^	2.70 × 10^−7^	kg/m^3^	Steel, chromium steel 18/8, hot rolled	{GLO}j market for j Cut-off, U
Cast Iron ^a^	6.50 × 10^−6^	kg/m^3^	Cast iron	{GLO}j market for j Cutoff, U
Galvanized Steel ^a^	2.20 × 10^−6^	kg/m^3^	Steel, low-alloyed, hot rolled	{GLO}j market for j Cutoff, U
Reinforced Concrete ^a^	2.90 × 10^−6^	m^3^/m^3^	Concrete, 25 MPa	{GLO}j market for j Cutoff, U
Asbestos Cement ^a^	6.60 × 10^−6^	kg/m^3^	Asbestos, crysotile type	{GLO}j production j Cut-off, U
Polyvinylchloride ^a^	2.50 × 10^−6^	kg/m^3^	Polyvinylchloride, emulsion polymerised	{GLO}j market for j Cutoff, U
Electricity ^b^	2.01 × 10^−1^	Kwh/m^3^	Electricity, medium voltage	{ZA}j market for j Cutoff, U
Tertiary Treatment				
Cast Iron ^a^	2.50× 10^−5^	kg/m^3^	Cast iron	{GLO}j market for j Cutoff, U
Stainless Steel 304 ^a^	2.00 × 10^−6^	kg/m^3^	Steel, chromium steel 18/8, hot rolled	{GLO}j market for j Cut-off, U
Reinforced Concrete ^a^	1.50 × 10^−4^	m^3^/m^3^	Concrete, 25 MPa	{GLO}j market for j Cutoff, U
Polyvinylchloride ^a^	1.00 × 10^−4^	kg/m^3^	Polyvinylchloride, emulsion polymerised	{GLO}j market for j Cutoff, U
Electricity ^b^	6.70 × 10^−2^	Kwh/m^3^	Electricity, medium voltage	{ZA}j market for j Cutoff, U
Chlorine ^a^	3.70 × 10^−3^	kg/m^3^	Chlorine, liquid	{GLO}j market for j Cutoff, U
Wastewater Transportation				
Diesel for WW transportation	2.44 × 10^−1^	kg/m^3^	Diesel	{GLO}j market group for j Cutoff, U
**OUTPUT**				
Chemical oxygen demand (COD) ^c^	1.85 × 10^−2^	kg/m^3^	Quality monitoring of foreground data from the centralized WWTP	
Nitrogen (N) ^c^	6.40 × 10^−3^	kg/m^3^	Quality monitoring of foreground data from the centralized WWTP	
Phosphorus (P) ^c^	7.50 × 10^−3^	kg/m^3^	Quality monitoring of foreground data from the centralized WWTP	
Methane (CH_4_) ^d^	8.00 × 10^−3^	kg/m^3^	Correlation background data	
Nitrus ocide (N_2_O) ^d^	3.38 × 10^−3^	kg/m^3^	Correlation background data	
Treated wastewater	1	m^3^/m^3^	Functional unit	
^a^ Mamathoni and Harding (2021) [17]; ^b^ Present study, ^c^ Haider et al. (2021) [1], ^d^ Campos et al. (2016) [33].				

## Appendix B. Life Cycle Inventory for Low-Cost Ceramic Membrane Bioreactor (LC-MBR)

**INPUT**	**Quantity**	**Units**	**Description**	**Ecoinvent 3.6 Market Process**
Primary treatment				
Cast Iron ^a^	3.20 × 10^−4^	kg/m^3^	Cast iron	{GLO}j market for j Cutoff, U
Mild steel ^a^	1.40 × 10^−4^	kg/m^3^	Steel, low-alloyed	{GLO}j market for j Cutoff, U
Stainless Steel 304 ^a^	3.60 × 10^−5^	kg/m^3^	Steel, chromium steel 18/8	{GLO}j market for j Cut-off, U
Polypropylene ^a^	3.20 × 10^−6^	kg/m^3^	Polypropylene, granulate	{GLO}j market for j Cutoff, U
Polyvinylchloride ^a^	2.80 × 10^−4^	kg/m^3^	Polyvinylchloride, emulsion polymerised	{GLO}j market for j Cutoff, U
Polyethylene ^a^	1.40 × 10^−5^	kg/m^3^	Polyethylene, high-density, granulate	{GLO}j market for j Cutoff, U
Reinforced Concrete ^a^	2.30 × 10^−7^	m^3^/m^3^	Concrete, 25 MPa	{GLO}j market for j Cutoff, U
Screen flush water ^a^	9.80 × 10^−2^	kg/m^3^	Tap water	{GLO}j market group for j Cutoff, U
Electricity ^b^	1.09 × 10^−1^	Kwh/m^3^	Electricity, medium voltage	{ZA}j market for j Cutoff, U
Secondary Treatment				
Reinforced Concrete ^a^	1.50 × 10^−6^	m^3^/m^3^	Concrete, 25 MPa	{GLO}j market for j Cutoff, U
Mild steel ^a^	7.20 × 10^−5^	kg/m^3^	Steel, low-alloyed	{GLO}j market for j Cutoff, U
Cast iron ^a^	7.90 × 10^−4^	kg/m^3^	Cast iron	{GLO}j market for j Cutoff, U
Polyvinylchloride ^a^	1.80 × 10^−6^	kg/m^3^	Polyvinylchloride, suspension polymerised	{GLO}j market for j Cutoff, U
Clay ^b^	3.04 × 10^−1^	kg/m^3^	Local clay	{GLO}j market for j Cutoff, U
Rice bran ^b^	7.61 × 10^−2^	kg/m^3^	Locally available rice bran	{GLO}j market for j Cutoff, U
NaOH for chemical cleaning of filter ^b^	2.28 × 10^−4^	kg/m^3^	NaOH, liquid	{GLO}j market for j Cutoff, U
Electricity ^b^	2.20 × 10^−1^	Kwh/m^3^	Electricity, medium voltage	{ZA}j market for j Cutoff, U
**Tertiary treatment**				
Reinforced Concrete ^a^	2.04 × 10^−4^	m^3^/m^3^	Concrete, 25 MPa	{GLO}j market for j Cutoff, U
Mild steel ^a^	1.37 × 10^−4^	kg/m^3^	Steel, low-alloyed	{GLO}j market for j Cutoff, U
Cast iron ^a^	3.30 × 10^−5^	kg/m^3^	Cast iron	{GLO}j market for j Cutoff, U
Polyvinylchloride ^a^	2.28 × 10^−6^	kg/m^3^	Polyvinylchloride, suspension polymerised	{GLO}j market for j Cutoff, U
Chlorine ^a^	4.00 × 10^−3^	kg/m^3^	Chlorine, liquid	{GLO}j market for j Cutoff, U
Electricity ^b^	3.27 × 10^−1^	Kwh/m^3^	Electricity, medium voltage	{ZA}j market for j Cutoff, U
**OUTPUT**				
Chemical oxygen demand (COD) ^c^	1.80 × 10^−3^	kg/m^3^	Quality monitoring of foreground data from a laboratory study	
Nitrogen (N) ^c^	3.00 × 10^−3^	kg/m^3^	Quality monitoring of foreground data from a laboratory study	
Phosphorus (P) ^c^	1.80 × 10^−4^	kg/m^3^	Quality monitoring of foreground data from a laboratory study	
Methane (CH_4_) ^d^	7.50 × 10^−3^	kg/m^3^	Correlation background data	
Nitrous oxide (N_2_O) ^d^	3.00 × 10^−3^	kg/m^3^	Correlation background data	
Treated wastewater	1	m^3^/m^3^	Functional unit	
^a^ Mamathoni and Harding (2021) [17]; ^b^ Present study, ^c^ Alresheedi et al. (2023) [9], ^d^ Campos et al. (2016) [33].				
